# Consumption of Ultra-Processed Foods and Health Harm

**DOI:** 10.3390/nu15132945

**Published:** 2023-06-28

**Authors:** Francesca Menichetti, Alessandro Leone

**Affiliations:** International Center for the Assessment of Nutritional Status and the Development of Dietary Intervention Strategies (ICANS-DIS), Department of Food, Environmental and Nutritional Sciences (DeFENS), University of Milan, 20133 Milan, Italy; francesca.menichetti@unimi.it

In recent decades, research has become increasingly interested in the relationship between diet and health. It is now well established that diet is a modifiable environmental factor that may be helpful to prevent many non-communicable diseases (NCDs) such as obesity, type 2 diabetes, cardiovascular diseases, and tumors. However, despite the proven benefits of a healthy diet, changes in food consumption have been observed. Food choices have shifted from the consumption of simple foods with established health benefits (e.g., whole grains, legumes, fish, nuts, fruit, and vegetables) to the consumption of unhealthy and ultra-processed foods (UPF), a hallmark of a Western diet [1,2].

According to a classification system used worldwide, the NOVA system, UPF are products formulated using five or more ingredients, including processed raw materials and additives, typically created using a range of industrial techniques and processes (e.g., carbonated beverages, pastries, cakes, ready-to-eat and ready-to-heat foods, packaged savory snacks, reconstituted meat products) [3]. These food products are under the magnifying glass of researchers and nutrition practitioners, as they are suspected of contributing to the development of obesity [4,5], cardiovascular diseases [6], diabetes (both type 2 and gestational diabetes) [7,8], and other NCDs.

This Special Issue entitled “Consumption of Ultra-Processed Foods and Health Harm” collected five articles and two reviews concerning the nutritional composition of UPF, and their effects on health.

Two papers aimed to quantify the daily consumption of UPF in different countries. Andarede et al. [9] reported that UPF contributed almost one-third (31.1%) of the total energy intake of French adults’ diet, particularly in young adults aged 18–39. They found that subjects with a higher consumption of UPF had a higher prevalence of inadequate energy, saturated fat, sugar and potassium intakes, as well as a deficient intake of fiber. This corroborated the poor nutritional quality of these products. A high variability among countries in the consumption of UPF has also been reported. A systematic review summarizing 99 articles regarding UPF consumption across 21 states worldwide revealed the highest consumption in the United States and the United Kingdom, where UPF account more than half of total energy intake (>50%), and the lowest intake in Mediterranean countries, especially in Italy (10%) [10]. Also, in this case, it has been underlined that young and adolescent people are more like to overconsume UPF [10].

The relationship between UPF consumption and their impact on health has also been explored. An article in this Special Issue [11] evaluated the relationship between UPF consumption and a decline in kidney function. Rey-Garcia et al. [11] reported that Spanish older adults who consumed higher amounts of UPF had a 50% greater risk of displaying a decline in kidney function than those with lower consumption. Moreover, Fliss-Isakow et al. [12] focused on the association between UPF, smoking and colorectal adenomas, confirming that unhealthy lifestyles, such as a diet rich in UPF and smoking, represent a health risk. In their study, smokers were found to be more vulnerable to the adverse effects of UPF and more likely to develop colorectal adenomas, regardless of type (nonadvanced, advanced) and location (distal, proximal). One more article, using different food classification systems, found an association between UPF consumption and many cardiometabolic risk factors [13].

However, health risks are not only related to the poor nutritional quality of UPF, but also to the presence of additives, which are increasingly being used to improve the stability and palatability of UPF, in order to make them more attractive to consumers. Their safety still needs to be clarified. Borsani et al. [14] investigated the impact on health of carrageenan (E-407), a food additive widely added in UPF for its properties as an emulsifier, gelling agent, thickener and stabilizer. In their review, they reported that persistent E-407 consumption may be related to microbiota dysbiosis and alteration of intestinal mucosal barrier, that can lead to inflammation and expression of pro-inflammatory molecules. Moreover, in clinical studies, E-407 may be related to allergic reactions and be responsible, in patients with inflammatory bowel diseases, for earlier relapse.

In light of this evidence, it is important to continue investigating the impact of UPF on health. To do this, it is essential to establish a universally recognized definition of UPF. Although the NOVA system is the best known and most widely used classification system, this is sometimes questioned. In fact, NOVA classification can be confusing because it is more related to the degree of food processing than to food nutritional quality. Martinez-Perez et al. [13], comparing four different UPF classification systems including NOVA, found different associations with cardiometabolic risk factors as a function of the classifications system used. Another article [15] in this Special Issue reported that 23% of foods classified as UPF according to the NOVA classification system had a high nutritional quality. This evidence supports the importance of standardizing criteria to define UPF. This is important to better analyze the adverse health effects of these products.

In summary, the studies presented in this Special Issue illustrate how the consumption of UPF has become widespread in recent years, particularly in some countries and group, and how their poor nutritional quality may negatively affect the health of consumers. Despite the lack of a universally recognized definition of UPF, these studies, as well as in the wider scientific literature at large, suggest the importance of limiting the consumption of industrially processed foods.
